# The effect of rituximab on encapsulated peritoneal sclerosis in an experimental rat model

**DOI:** 10.3906/sag-1911-13

**Published:** 2020-06-23

**Authors:** Süleyman KARAKÖSE, Ayşe Zeynep BAL, Eylem Pınar ESER, Murat DURANAY

**Affiliations:** 1 Department of Nephrology, Konya Training and Research Hospital, Konya Turkey; 2 Department of Nephrology, Ankara Training and Research Hospital, Ankara Turkey; 3 Department of Pathology, Ankara Training and Research Hospital, Ankara Turkey

**Keywords:** Rituximab, encapsulated peritoneal sclerosis, matrix metalloproteinase-2, transforming growth factor-beta, vascular endothelial growth factor

## Abstract

**Background/aim:**

Peritoneal sclerosis may be observed in varied manifestations. However, the most serious form is the encapsulated peritoneal sclerosis. We researched the effect of rituximab on peritoneal fibrosis in an experimental rat model.

**Materials and methods:**

Twenty-four Wistar Albino rats were divided into 4 equal groups. During weeks 0–3; group I received isotonic saline (IS) solution, group II, group III, and group IV received chlorhexidine gluconate (CG) via intraperitoneal (i.p.) route. In the next 3 weeks nothing adminestred to both group I and group II but IS solution was adminestred to group III via i.p. route and 375 mg/m2/week rituximab was applied intravenously on days 21, 28, and 35 to group IV. Fibrosis, peritoneal thickness, and inflammation were evaluated. Immunohistochemical methods used for the detection of matrix MMP-2, TGF-β1, and VGEF expressions.

**Results:**

The rituximab (group IV) had significantly lower fibrosis and peritoneal thickness scores than the group II and III (P < 0.001). TGF-β1 and VEGF expressions were significantly lower in the rituximab group than in the group II and III (P < 0.001).**Conclusion**: We found that rituximab had a significant effect on the peritoneal thickness, total fibrosis, TGF-β1 and VGEF scores which were induced by CG.

## 1. Introduction

Peritoneal sclerosis may be observed in varied manifestations. However, the most serious form is encapsulated peritoneal sclerosis (EPS). EPS has a low prevalence of up to 3%, but it is associated with a high mortality rate of up to 51% in patients undergoing peritoneal dialysis (PD). Therefore, EPS is of major concern to nephrologists []. The characteristic feature of EPS is extreme sclerosis of the peritoneal membrane, which covers and constricts the intestines []. Although several studies have examined EPS, the exact pathophysiology of EPS remains unclear. The triggering factors involved in the pathogenesis of systemic fibrosis and EPS were M2-type macrophages, CD4+T cells [], B cells [], the matrix metalloproteinase (MMP) family, particularly MMP-2 [,], and transforming growth factor-beta 1 (TGF- β1) [].

Nishino et al. reported that T and B lymphocytes had important roles in the process of peritoneal fibrosis in a mouse peritoneal fibrosis model []. However, Habib et al. reported that there were no CD20 and CD15 positive cells in the biopsies of a subgroup of patients with EPS []. Conversely, Bosello reported the potential role of B cells in tissue fibrosis in some experimental models, thus, targeting B cells could be one method of reducing extracellular matrix deposition and lowering the inflammatory status [4]. Research has also shown the effect of the anti-B cell monoclonal antibody, rituximab, in the treatment of diseases involving fibrotic processes [4,,].

A variety of therapeutic approaches to EPS including surgical and medical options have been reported []. In recent studies, the effects of various immunosuppressive drugs such as prednisone, azathioprine, mycophenolate mofetil (MMF), and sirolimus have been investigated [,].We reported the effect of the T cell blocker abatacept in the treatment of EPS in our previous study []. However, to our knowledge, no data on the effect of the anti-CD 20+ antibody, rituximab, in EPS models are available. Rituximab (MabThera/Rituxan), a chimeric murine/human monoclonal antibody, binds specifically to the transmembrane antigen CD20 on B cells []. 

The aim of this study was to investigate the effect of rituximab in an experimental rat model in which chlorhexidine gluconate was used to induce peritoneal fibrosis.

## 2. Materials and methods

The Institutional Animal Use and Care Committee of the Ankara Education and Research Hospital approved the study protocol, and the study was performed in accordance with the National Institutes of Health guidelines. Twenty-four female Wistar Albino rats with a mean weight of 180–200 g were selected for the study. The rats were randomly divided into 4 equal groups and kept at room temperature (24°C) in a 12-h light/dark cycle in polycarbonate cages and fed a standard laboratory diet for 42 days. The EPS model was performed as described by Ishii et al. [].

During weeks 0–3, group I (control group) received isotonic saline (IS) (2 mL/day) solution intraperitoneally (i.p.), group II (CG group), group III (CG + IS group), and group IV (rituximab group) received chlorhexidine gluconate (CG) solution (2 mL of 0.1% CG and 15% ethanol dissolved in IS) via the i.p. route. In the next 3 weeks, nothing was administered to both group I and group II, but IS solution was administered to group III and 375 mg/m2/week rituximab (MabThera) (diluted with saline to 1mg/mL) was given intravenously on days 21, 28, and 35 to group IV. A 23-G needle was used for all intra abdominal injections. In order to eliminate the effects of recurrent injections to the peritoneum, daily injections were administered to the lower part of the abdominal peritoneal cavity, whereas for the pathologic investigations, the right-left upper quadrant of the parietal peritoneum was preferred.

### 2.1. Histologic examination

Ketamine and xylazine were used for anesthesia, and on the 42nd day of the study, all rats were euthanized via cervical dislocation. Following laparotomy, parietal peritoneal samples were collected from the right-left upper quadrant of the abdomen. The peritoneal membrane samples were fixed in 4% formalin and embedded in paraffin wax. Paraffin blocks were cut into 5-μm-thick sections and stained using hematoxylin–eosin (HE) and Masson’s trichrome (MT). A pathologist, who was blinded to the treatment groups, examined the samples. The peritoneal thickness, fibrosis, degree of inflammation; and TGF-β1, MMP-2, VGEF scores were investigated in all groups. An ocular micrometer was used to measure the peritoneal thickness. Measurements were performed for 3 areas, and the mean value was considered as the peritoneum thickness.

The pathologist semiquantitatively scored the degree of fibrosis in MT-stained sections on a 5-point scale: score 0 = none; 1 = low; 2 = mild; 3 = moderate; 4= high moderate; and 5 = severe. The scoring system for 3 parameters that described the fibrosis score was as follows: the subserosal fibrotic matrix (on a 2-point scale: 0 = none; 1 = mild; and 2 = marked), subserosal large collagen fibers (on a scale of 0–1: 0 = absent and 1 = present), and subserosal fibrotic proliferation (on a 2-pointscale: 0 = none; 1 = mild, and 2 = intense) [16]. The fibrosis score was calculated by summing the subserosal large collagen fibers, subserosal fibrotic proliferation, and subserosal fibrotic matrix scores. The degree of inflammation in HE-stained sections was scaled as score 0 = none; 1 = mild inflammation (presence of few scattered inflammatory cells); 2 = moderate inflammation (small groups of inflammatory cells in high-power fields); and score 3 = severe inflammation (many inflammatory cells in either a diffuse pattern or large groups). Vasculopathy was evaluated as described by Williams et al. [].

### 2.2. Immunohistochemical staining

Anti-MMP-2, anti-TGF-β1, and anti-VGEF antibodies were used for the immunostaining of MMP-2, TGF-β1, and VGEF using the Leica Bond-Max automatic immunostainer (Novocastra-Leica Microsystems, Newcastle upon Tyne, UK). Deparaffinized sections were treated with 0.3% hydrogen peroxide (H2O2) and blocked with 0.5% bovine serum albumin for 10 min at room temperature. The sections were incubated with anti-MMP-2 antibody (mouse monoclonal, 1:50 diluted), anti-TGF-β1 antibody (mouse monoclonal, 1:100 diluted), and anti-VGEF antibody (mouse monoclonal, 1:200 diluted) for 20 minutes, followed by a further incubation with 0.1% H2O2 for 10 minutes. Hematoxylin was used as a counterstain. Positive staining was scored in a semiquantitative manner from 0 to 4:0 = almost no staining; 1 = staining < 25%; 2 = staining 25–50%; 3 = staining 50–75 %; and 4 = staining > 75% [].

### 2.3. Statistical analyses

The SPSS statistical package, version 24.0 (IBM Corp., Armonk, NY, USA), for Windows was used for the statistical analyses. Shapiro–Wilk test was performed to determine the normality of distribution. Descriptive statistics were presented as mean ± SEM for normally distributed variables and as median (range) for nonnormally distributed variables. The significance of the difference of the means between the groups were evaluated by the one-way ANOVA test for peritoneal thickness. If there was a significant difference between the groups, a post-hoc analysis was performed to determine which group had a different result. Comparison of nonnormal distributed variables (Inflammation, Total Fibrosis, MMP 2, TGF beta, and VEGF scores) was made by Kruskal–Wallis test. Kruskal–Wallis multiple comparison test was performed to the significance of pairwise differences using Bonferroni correction to adjust for multiple comparisons. 

## 3. Results

**Figure 1:**
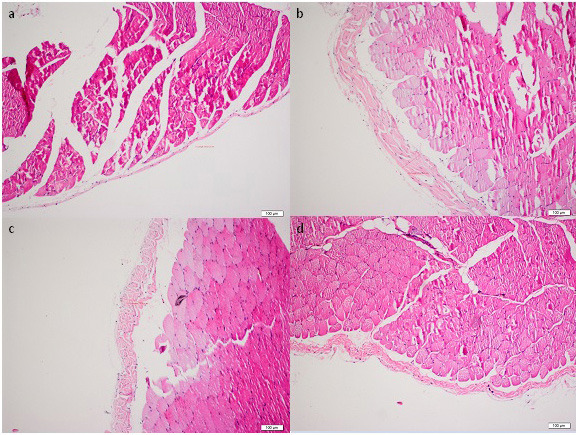
HE staining of parietal peritoneal tissue samples of all groups (× 100). (a: Group I, b: Group II, c: Group III, d:Group IV)

**Figure 2:**
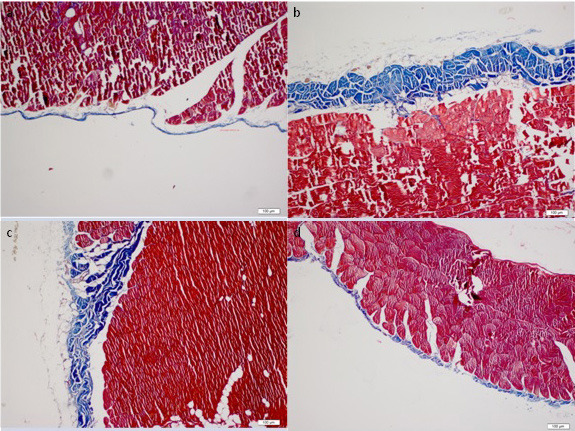
Masson’s trichrome staining of the parietal peritoneal tissue samples of all groups (× 100). (a: Group I, b: Group II, c: Group III, d:Group IV)

**Figure 3:**
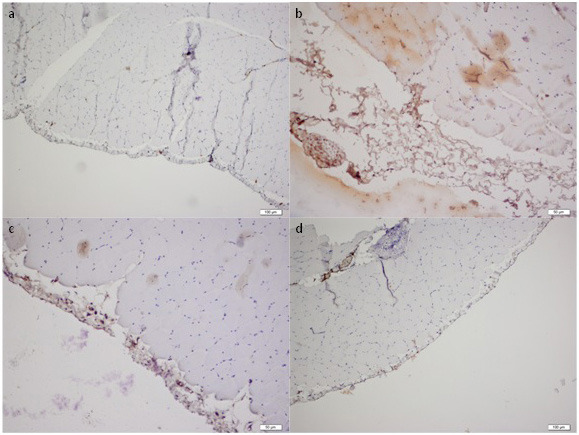
Immunohistochemical analysis of MMP-2 expressions in the peritoneal tissue samples of all groups (× 10 ihk). (a: Group I, b: Group II, c: Group III, d:Group IV)

**Figure 4:**
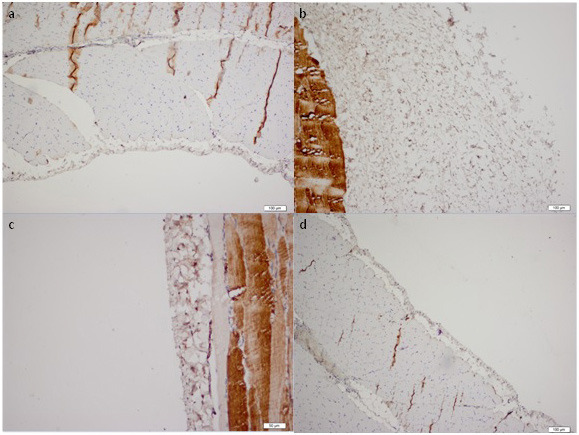
Immunohistochemical analysis of the TGF-β1 expression in the peritoneal tissue samples of all the groups (× 10 ihk). (a: Group I, b: Group II, c: Group III, d:Group IV)

**Figure 5:**
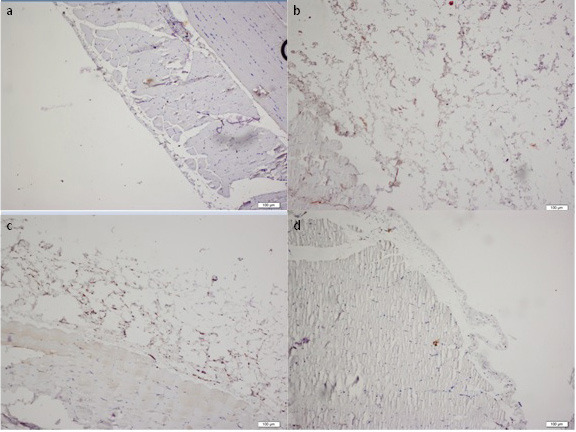
Immunohistochemical analysis of theVGEF expression in the peritoneal tissue samples of all groups (× 10 ihk). (a: Group I, b: Group II, c: Group III, d:Group IV)

Throughout the study all the animals have stable weight and health, and rituximab was well tolerated without an adverse event. The histopathological findings of the peritoneum are demonstrated in Table. Morphological parameters, including peritoneal thickness, inflammation, and fibrosis scores of the group II were significantly different from those of the group I (1761.5 ± 834.9 μm vs. 261.7 ± 104.5 μm, 1.5 (IR:1.75) vs. 0 (IR:0.75) and 4 (IR:1.75) vs. 0.5 (IR:1), respectively; P < 0.05). Significantly better histopathological parameters in terms of peritoneal thickness and fibrosis scores were observed in the rituximab group than in the group III (334.8 ± 126.1 vs. 2518.1 ± 1401 μm and 1 (IR:0.75) vs. 4 (IR:2), respectively; P < 0.05). However, the inflammation score in group IV was not statistically different from group II and III [1 (IR:1) vs. 1.5 (IR:1.75) and 1 (IR:1) respectively; P > 0.05]. The group II and III had similar morphological parameter results (Table). There was no difference in terms of vascular scores between groups. Parietal peritoneal tissue samples of all groups were assessed using 2 different histological methods, HE staining (Figure 1) and MT staining (Figure 2). MMP-2, TGF-β1, and VGEF scores of parietal peritoneum are also summarized in Table. The group II and III had higher tissue MMP-2, TGF-β1, and VGEF scores than the group I. The TGF-β1 and VGEF scores level were significantly lower in group IV than group III. The immunohistochemical staining of parietal peritoneal tissue samples of all groups with MMP-2, TGF-β1, and VGEF antibodies are shown in Figures 3, 4, and 5.

**Table 1:** Histopathologic parameters of the peritoneum and MMP-2, TGF-β, and VGEF results in each group.

Mean ± SD Median (IR)	Control (Group I)	CG (Group II)	CG+SF (Group III)	CG+Rituximab (Group IV)	P
Peritoneal thickness (μm)	261.7 ± 104.5	1761.5 ± 834.9a	2518.1 ± 1401.3a	334.8 ± 126.1b,c	<0.001
Inflammation score (0–3 scale)	0 (0.75)	1.5 (1.75)a	1(1)a	1 (1)	0.008
Total Fibrosis score (0–5 scale)	0.5 (1)	4 (1.75)a	4 (2)a	1 (0.75)b,c	<0.001
MMP 2 score	0 (1)	2 (1.75 )a	2 (2)a	1 (1)	0.001
TGF beta score	0 (1)	1.5 (1)	2.5 (1.75)a	0.5 (1)b	0.001
VEGF score	0 (0.75)	1 (1)	2 (1)a	0 (0.75)b	0.001

a P < 0.05 as compared with control b P < 0.05 as compared with CG+SF c P < 0.05 as compared with CG

## 4. Discussion

We demonstrated the effectiveness of rituximab in lowering fibrosis scores and peritoneal thickness in an experimental rat model. Although the inflammation score in the rituximab group was lower than in group II, the difference was not statistically significant. However, the fibrotic marker TGF-β1, as well as the vascular factor VEGF were significantly lower in the rituximab group than group III.

EPS could be accepted as an orphan disease because its pathogenesis and treatment remains unclear. Bowel rest with total parenteral nutrition, surgical interventions, and a few drugs that have been used in patients have been proposed for EPS treatment [,]. Immunosuppressive drugs, such as everolimus, MMF, abatacept, and thalidomide, have been tested on experimental rat models and demonstrated to be effective in EPS treatment [,,10,].

Several studies have reported that B cells play a pathogenic role in diseases with fibrotic processes [,,,,]. Activated B cells and macrophages might secrete high amounts of TGF-β, which have important roles in the pathogenesis of fibrosis []. Nishino et al. [8] investigated the role of lymphocytes in the process of peritoneal fibrosis in an experimental rat model and showed that the thicker submesothelial compact zone had a higher amount of B lymphocytes and macrophages than those observed in control immune deficient and wild-type mice.

However, the effect of immune modulator agents that block B cells in EPS treatment remain unknown. To our knowledge, this is the first study that investigate the effect of rituximab on peritoneal fibrosis. Rituximab significantly reduced peritoneal thickness and fibrosis in our study. Peritoneal thickness, fibrosis scores, as well as the TGF-β1 and VGEF scores were significantly lower in group IV than in group III. When compared with our previous study with abatacept, rituximab was more effective in improving peritoneal thickness and fibrosis. However, abatacept was more effective in improving the inflammation score [15].

Studies with sirolimus and everolimus showed similar results to rituximab; each decreased peritoneal thickness and fibrosis but had not significant effect on inflammation scores [,]. Huddam et al. reported that MMF had a significant effect on fibrosis, peritoneal thickness, and inflammation scores. However, the MMP-2 level increased after MMF treatment in their study [23]. Another study showed that after MMF treatment, MMP-2 and TGF-β1 gene expressions decreased significantly []. Moreover, sirolimus showed no significant effect on MMP-2 levels [31].

MMF and sunitinib treatment have a significant impact on dialysate TGF-β1 levels as compared with the resting EPS rat model [,]. The tissue immunohistochemical score of TGF-β1 significantly decreased after the rituximab treatment in our study. This is the first study to investigate the tissue immunohistochemical score of VGEF. The VGEF score was significantly lower in group IV than in group III.

Consequently, we demonstrated that rituximab might reduce fibrosis and peritoneal thickness, and TGF-β1, VGEF scores in an EPS rat model. For evidence-based treatment of EPS, further larger and detailed studies are required to show the effectiveness of rituximab.

## Acknowledgements

The study was approved by the Institutional Animal Care, Use and Ethics Committee (project number: 2017/0039-471, date: 26.05.2017). No funding is received for this study. 

## Conflict of Interest

The authors declare that there is no conflict of interest.
